# Supplementary material for: The First Exploratory Personalized Medicine Approach to Improve Bariatric Surgery Outcomes Utilizing Psychosocial and Genetic Risk Assessments: Encouraging Clinical Research
Source: J Pers Med. 2023 Jul 20;13(7):1164. doi: 10.3390/jpm13071164 (PMC10381606; doi:10.3390/jpm13071164)
Supplement: Supplementary file 1 [file jpm-13-01164-s001.zip › jpm-2410298-supplementary.pdf]

Supplemental Materials

Supplemental Table S1: Psychosocial Questionnaire Inventories

Inventory Name	*Number of Items*	*Item Rating Scale*	*Reference**
Food Cravings Questionnaire – Trait Reduced (FCQ-T)	15	1-6	Meule, A., Hermann, T., & Kubler, A. 2014. A short version of the Food Cravings Questionnaire-Trait: the FCQ-T-reduced. *Frontiers in Psychology*, 5, 190.
Modified Yale Food Addiction Scale 2.9 (mYFAS 2.0)	13	0-7	Gearhardt, A., White, M., Masheb, R., Morgan, P., Crosby, R., Grilo, C. 2012. An examination of the Food Addiction Construct in obese patients with binge eating disorder. *Int J Eat Disord*, 45(5): 657-663.
Eating Expectancies Inventory (EEI)	34	1-7	Fitzsimmons-Craft, E., Keatts, D., & Bardone-Cone, A. 2013. Eating expectancies in relation to eating disorder recovery. *Cognit Ther Res*. 37(5): 104.
Eating Attitudes Test -26 (EAT-26)	26	0-3	DM Garner, et al. The Eating Attitudes Test: Psychometric Features and Clinical Correlates. 12 Psychological Medicine 871-878. 1982.
Quality of Life Enjoyment and Satisfaction Questionnaire- Short form (QLES-Q-SF)	14	1-5	Stevanovic, D. 2011. Quality of life enjoyment and satisfaction questionnaire-short form for quality of life assessments in clinical practice: a psychometric study. Journal of Psychiatric and Mental Health Nursing, 18(8).
Chronic Stress Index (CSI)	22	1-5	Schulz AJ, Mentz G, Lachance L, Johnson J, Gaines C, Israel BA. Associations between socioeconomic status and allostatic load: effects of neighborhood poverty and tests of mediating pathways. Am J Public Health. 2012;102(9):1706–14.
Difficulties in Emotion Regulation Scale – Short Form (DERS-SF)	18	1-5	Kaufman, E., Xia, M., Fosco, G., Yaptangco, M., Skidmore, C., Crowell, S. 2015. The Difficulties in Emotion Regulation Scale Short Form (DERS-SF): Validation and replication in adolescent and adult samples. J Psychological Behav Assess, 37(3).
The Center for Epidemiologic Studies Depression Scale (CESDS)	20	0-4	Kapadia, S.G., Wei, C., Bartlett, S.J., Lang, J., Wise, R.A., Dixon, A.E. 2014. Obesity and symptoms of depression contribute independently to the poor asthma control of obesity. *Respiratory Medicine*, 108*:* 1100-1107.
Weight Influenced Self-Esteem Questionnaire (WISE-Q)	22	0-4	Trottier K, McFarlane T, Olmsted MP, McCabe RE. 2013. The Weight Influenced Self-Esteem Questionnaire (WISE-Q): factor structure ad psychometric properties. *Body Image* 10:112-20
Pittsburgh Sleep Quality Index (PSQI)	18	0-3	Buysse DJ, Reynolds CF, Monk TH, Berman SR, Kuper DJ: The Pittsburgh Sleep Quality Index: A new instrument for psychiatric practice and research. Psychiatry Research 28:193-213, 1989

Additional information and scoring instructions previously described in referenced literature.

Supplemental Table S2: GARS Panel*

Gene	Polymorphism	Location	Risk Allele(s)
Dopamine D1 Receptor DRD1	rs4532 SNP	Chr 5	A
Dopamine D2 Receptor DRD2	rs1800497 SNP	Chr 11	A
Dopamine D3 Receptor DRD3	rs6280 SNP	Chr 3	C
Dopamine D4 Receptor DRD4	rs1800955 SNP<br>48 bases Repeat VNTR	Chr 11<br>Chr 11, Exon 3	C<br>7R,8R,9R,10R,11R
Catechol-O-Methyltransferase COMT	rs4680 SNP	Chr 22	G
Mu-Opioid Receptor OPRM1	rs1799971 SNP	Chr 6	G
Dopamine Active Transporter DAT 1	40 bases Repeat VNTR	Chr 5, Exon 15	3R,4R,5R,6R,7R,8R
Monoamine Oxidase A MAOA	30 bases Repeat VNTR	Chr X, Promoter	3.5R, 4R
Serotonin Transporter SLC6A4 (5HTTLPR)	43 bases Repeat INDEL/VNTR plus rs25531 SNP	Chr 17	LG, S
GABA(A) Receptor, Alpha-3 GABRB3	CA-Repeat DNR	Chr 15 (downstream)	181

Table adopted from Blum et al., 2020. [13]
